# The Role of Adipose Tissue in the Pathogenesis and Therapeutic Outcomes of Inflammatory Bowel Disease

**DOI:** 10.3390/cells8060628

**Published:** 2019-06-21

**Authors:** Piotr Eder, Maciej Adler, Agnieszka Dobrowolska, Julian Kamhieh-Milz, Janusz Witowski

**Affiliations:** 1 Department of Gastroenterology, Dietetics and Internal Medicine, Poznań University of Medical Sciences, Heliodor Święcicki Hospital, 60-355 Poznan, Poland; agdob@ump.edu.pl; 2 Department of Paediatrics, Royal Berkshire Hospital NHS Foundation, RG1 5AN Reading, UK; maciej.adler11@imperial.ac.uk; 3 Institute for Transfusion Medicine, Charité–Universitätsmedizin Berlin, 10117 Berlin, Germany; julian.milz@charite.de; 4 Department of Pathophysiology, Poznań University of Medical Sciences, 60-806 Poznan, Poland; jwitow@ump.edu.pl

**Keywords:** inflammatory bowel disease, mesentery, adipose tissue, inflammation

## Abstract

Though historically regarded as an inert energy store, adipose tissue is a complex endocrine organ, which is increasingly implicated in the pathogenesis of inflammatory bowel disease (IBD). Accumulating evidence points to visceral adipose tissue and specifically to its mesenteric component, or “creeping fat” as impacting on the disease course through its immunomodulatory properties. On the one hand, mesenteric fat acts as a physical barrier to inflammation and is involved in controlling host immune response to translocation of gut bacteria. On the other hand, however, there exists a strong link between visceral fat and complicated course of the disease with unfavorable therapeutic outcomes. Furthermore, “creeping fat” appears to play different roles in different IBD phenotypes, with the greatest pathogenetic contribution probably to an ileal form of Crohn’s disease. In this review, we summarize and discuss the existing literature on the subject and identify high-priority areas for future research. It may be that a better understanding of the role of mesenteric fat in IBD will determine new therapeutic targets and translate into improved clinical outcomes.

## 1. Introduction

Inflammatory bowel disease (IBD) encompasses Crohn’s disease (CD) and ulcerative colitis (UC), both of which are characterized by chronic recurrent inflammation and frequent intestinal and extra-intestinal complications. IBD is thought to develop in genetically susceptible individuals as a result of dysregulated mucosal response to commensal gut bacteria. However, the exact pathogenetic mechanisms involved in IBD are not fully elucidated [1]. Population studies show an increasing burden of IBD [2,3], although with a variable worldwide distribution [4]. Mirroring global trends, the prevalence of obesity in the population of IBD patients is also rising [5,6]. As a result, the potential involvement of adipose tissue in intestinal inflammation has gained increasing attention. Moreover, there is a growing body of evidence suggesting that adipose tissue can affect disease progression, prognosis and therapeutic outcomes. Here, we summarize the available data on the role of adipose tissue in the pathogenesis of IBD and the relationship between certain features of adipose tissue and the efficacy and safety of therapeutic interventions for IBD.

## 2. Adipose Tissue and the Pathogenesis of IBD

**Figure 1:**
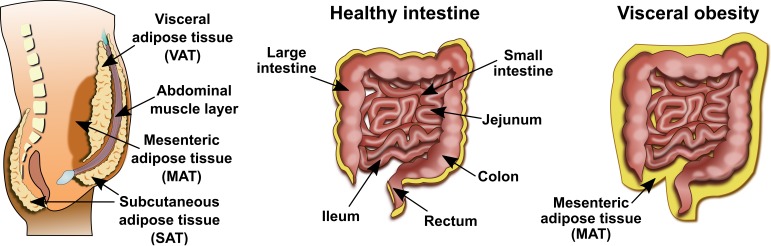
Classification of adipose tissue according to its anatomical location and distribution of visceral fat in health and obesity.

Adipose tissue is characterized by substantial structural and functional heterogeneity [7]. Anatomically, adipose tissue is classified into subcutaneous (SAT) and internal adipose tissue, which encompasses visceral (intra-thoracic and intra-abdominal) and non-visceral (intra- and peri-muscular) adipose tissue (Figure 1).

The vast majority of data on the role of adipose tissue in IBD relates to SAT and intra-abdominal visceral adipose tissue (VAT). Only few studies have specifically addressed the role of mesenteric adipose tissue (MAT), a peri-intestinal compartment of VAT. In the subject literature, the terms VAT and MAT have been occasionally (though imprecisely) used synonymously. In this review, we will attempt to distinguish between the two terms where possible, as MAT appears to play a particularly important role in IBD.

### 2.1. IBD and Adiposity-Shortcomings of BMI

There are a number of plausible biological mechanisms underlying the interaction between obesity and IBD. First, adipose tissue is an important source of proinflammatory cytokines including tumor necrosis factor-alpha (TNFα), interleukin-(IL)-6 (IL-6) and IL-8 (CXCL8), which contribute to obesity-associated ‘smoldering’ inflammation [7]. However, there is no straightforward association between body mass index (BMI) and severity of IBD [8,9]. It has been shown that the prevalence of obesity in IBD patients is similar to that in the general population [10]. Curiously, obesity in IBD seems to be associated with a milder course of the disease [11]. A recent meta-analysis of seven studies with a pooled population of 16,220 patients showed that obese patients were less likely to undergo surgery, be hospitalized and use corticosteroids [12]. However, obesity did not seem to be associated with the presence of perianal lesions, or response to treatment with immunomodulators or anti-TNFα agents [12]. These inconsistencies may be related to significant limitations of BMI as a biomarker of adiposity [13,14], as it is unable to differentiate between subcutaneous and visceral adipose tissue [15]. Moreover, standard BMI cut-off values for obesity have not been validated in IBD patients [16]. In fact, lower BMI values in patients with aggressive IBD may be result from malnutrition that accompanies severe inflammation.

Considering different adipose tissue compartments, it appears that MAT or VAT have the main influence on the IBD course. Although SAT covers approximately 80% of the total body fat, it is involved mainly in controlling the caloric balance and—as such—plays a very important role in the pathogenesis of metabolic disorders including diabetes or non-alcoholic fatty liver disease [16]. In IBD, changes in the SAT volume can modulate the pharmacokinetics of several drugs; however, the data in this respect are inconsistent. Increased SAT has been shown to worsen therapeutic outcomes by decreasing 6-thioguanine and adalimumab levels and by accelerating the loss of response to infliximab. Another clinical implication is the higher rate of perioperative morbidity in obese patients undergoing surgery for both IBD-related and IBD-unrelated conditions [16]. Interestingly, although the SAT volume can decrease in severe cases of IBD due to malnutrition, the number of patients with an increase in SAT is still high. This is a consequence of the obesity epidemic in the 21^st^ century, whose hypothetical influence on the IBD course is still poorly understood.

### 2.2. Mesenteric Adipose Tissue in IBD: A Unique Environment and A Double-Edged Sword

As previously discussed above, mounting evidence suggests that visceral rather than subcutaneous fat plays a role in CD. The phenomenon of inflammatory mesenteric fat hypertrophy, or “creeping fat”, was first described by Burril Crohn in 1932 [17]. It is defined as the expansion of white adipose tissue from the mesentery towards the intestine, resulting in partial coverage of the intestine and loss of the bowel-mesentery angle [18]. Intraoperatively, it demarcates the most severe lesions, setting the margins for resection [19].

Interestingly, the reach of mesenteric “creeping fat” directly matches the extent of transmural lesions, and the mesenteric and mucosal transition zones, seen macroscopically, correspond with each other [19]. The development of mesenteric “creeping fat” in CD has been hypothesized to be caused by adipocyte hyperplasia rather than hypertrophy [20], resulting in an approximately four-fold increase in the number of mesenteric adipocytes compared with healthy controls [21]. In addition to adipocytes (or pre-adipocytes), MAT in IBD also consists of macrophages, fibroblasts, extracellular matrix and abundant vasculature [22].

**Figure 2:**
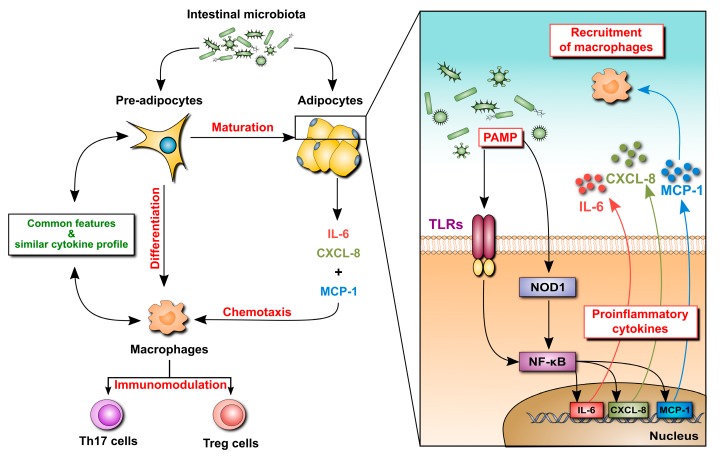
Potential mechanisms underlying the involvement of mesenteric adipocytes in the intestinal inflammatory response in inflammatory bowel disease (IBD). Pre-adipocytes in the mesenteric fat respond to translocated intestinal bacteria by sensing microbe-derived molecules (pathogen-associated molecular patterns, PAMP) with pattern recognition receptors, such as toll-like receptors (TLRs) or nucleotide oligomerization domain receptor-1 (NOD1). The resulting signaling cascades lead to activation of transcription factors (such as NF-kappa B) and induction of genes for proinflammatory cytokines and chemokines. This leads to adipose tissue infiltration by leukocytes, including macrophages that modulate local inflammation and immune response. In addition, pre-adipocytes can differentiate into macrophages further driving the inflammatory reaction.

The function of MAT during IBD is not entirely clear [23]. MAT is thought to create a reactive immunological zone around the inflamed intestine [24,25]. The gastrointestinal epithelial barrier is critical for maintaining the equilibrium between commensal microbiota and the host’s immune system [26,27,28]. As a result of impaired epithelial integrity, transmural inflammation in CD enables the bacteria to translocate into the mesentery [29]. Both pre-adipocytes and adipocytes express functional pattern recognition receptors, such as toll-like receptors (TLRs) [30,31,32,33] and nucleotide oligomerization domain receptors (NODs) [34], which respond to bacteria-derived molecules by releasing proinflammatory mediators (Figure 2) [35,36]. In vitro stimulation of mature adipocytes with a NOD-1 specific ligand activates nuclear factor—kappa B (NF-kB) transcription factor and induces the production of proinflammatory cytokines, including monocyte chemoattractant protein-1 (MCP-1), IL-6 and IL-8 [37]. Crucially, NOD-1 and NOD-2 receptors have been identified as CD susceptibility genes [38,39]. Increased expression of MCP-1 by adipocytes leads to tissue infiltration by macrophages [23]. Pre-adipocytes can differentiate into macrophages and have many features in common (Figure 2) [40]. These include similar profiles of gene expression and proinflammatory cytokine release, as well as phagocytic activity [40,41].

Interestingly, there are some data pointing to a protective rather than injurious role of MAT in IBD. By promoting local inflammation, the activated adipose tissue supports local host defense, limiting systemic inflammation and reducing the risk of perforation [42]. In this respect, leptin, which induces TLR expression in pre-adipocytes and adipocytes [43], has been observed to be upregulated locally but not systemically in IBD [44,45]. Kredel et al. investigated in vivo effects of leptin and adiponectin on M1 and M2 macrophages in “creeping fat” [24]. Leptin and adiponectin receptors were expressed uniformly by both macrophage subgroups and neither leptin nor adiponectin affected macrophage polarization. Nevertheless, leptin and adiponectin appeared to induce a stronger response in M2 macrophages, leading to the increased production of anti-inflammatory IL-10. Furthermore, adiponectin affected predominantly M2 macrophages to produce chemotactic activity towards T cells [24]. This unique inflammatory environment found in hypertrophied mesenteric fat supports the view that VAT acts as a fourth barrier in the local host defense system in addition to the intraluminal mucus, the epithelial monolayer and the lamina propria [42].

The majority of studies on “creeping fat” in IBD related the observed effects to those in healthy non-obese controls. More recently, however, Zulian et al. compared CD patients to non-CD individuals with obesity in terms of morphology and gene expression profiles in various fat deposits [46]. Compared with healthy non-obese controls, the authors report that the expression of proinflammatory genes in VAT (e.g., *STAT1, STAT4, ICAM2, IL-8, CCL2, VCAM1*) was significantly increased both in CD patients and in non-CD obese individuals. Interestingly, the proportion of anti-inflammatory genes expressed by VAT was higher in CD than in simple obesity. These observations were partially supported by Coope et al. [47]. The authors assessed transcriptional and molecular pathways activated in MAT in CD in comparison with non-IBD controls. They showed an increase in IL-10 expression, accompanied by a decrease in NF-κB pathway activation, reflected by a decreased pIκB/IκB ratio, suggesting a potential anti-inflammatory role of MAT. On the other hand, increased expression of the signal transducer and activator of transcription 1 (STAT1) may reflect the activation of proinflammatory pathways in MAT [47]. These complex observations support the proposed role of “creeping fat” as both the target and the regulator of inflammation in IBD.

### 2.3. Mesenteric Adipose Tissue: Dissimilarities between IBD Phenotypes

Traditionally, IBD has been separated into UC and CD, with further subgroups defined by the Montreal Classification [48]. However, in as many as 15% of patients, the disease shows overlapping features and cannot be precisely classified [49]. Moreover, recent genomic association studies revealed the presence of significant differences between ileal and colonic CD, with the latter placed genetically between ileal CD and UC [50]. Therefore, it may well be that the role and significance of mesenteric adiposity differs across IBD phenotypes.

Kredel et al. [51] assessed intestinal mucosa and MAT from patients with ileal CD, colonic CD and UC. The phenomenon of “creeping fat” appeared to be restricted to ileal specimens, and was less prominent in colonic CD and UC, with the latter showing no circular behavior and the presence of hypertrophy only in the epiplotic appendices. Moreover, “creeping fat” in the ileum contained significantly more fibrotic tissue and T cells than colonic fat from CD or UC patients. Immunotyping of T cells revealed a higher proportion of Treg cells in the ileal versus colonic adipose tissue. The fraction of Th17 cells was greater in the mucosa of ileal CD patients and correlated negatively with clinical activity of the disease (as assessed by the Crohn’s Disease Activity Index, CDAI). Moreover, the percentage of Th1 cells was significantly higher in MAT than in the mucosa of all IBD groups. Remarkably, colonic fat from CD patients shared features of both ileal fat from CD patients and colonic fat from UC patients, supporting the concept that these entities should be considered separately.

This view is further reinforced by recent studies on the interaction between gut microbiome and the MAT. The advent of culture-independent microbiome profiling has greatly advanced our understanding of the intestinal flora in health and IBD [52,53]. Using next generation sequencing, Kiernan et al. [54] analyzed the microbiota in the mesenteric lymph nodes from IBD patients undergoing bowel resection. They found significant differences between CD and UC patients, with CD characterized by the overexpression of *Proteobacteria* (a phylum containing such pathogens as *E. coli, Shigella, Salmonella and Helicobacter spp*.). Moreover, the ratio of *Firmicutes-*to-*Bacteroides* was found to be decreased in CD but increased in UC. Curiously, the microbial profile of a given patient was consistent and independent of the sampling location and/or the presence of local inflammation.

**Figure 3:**
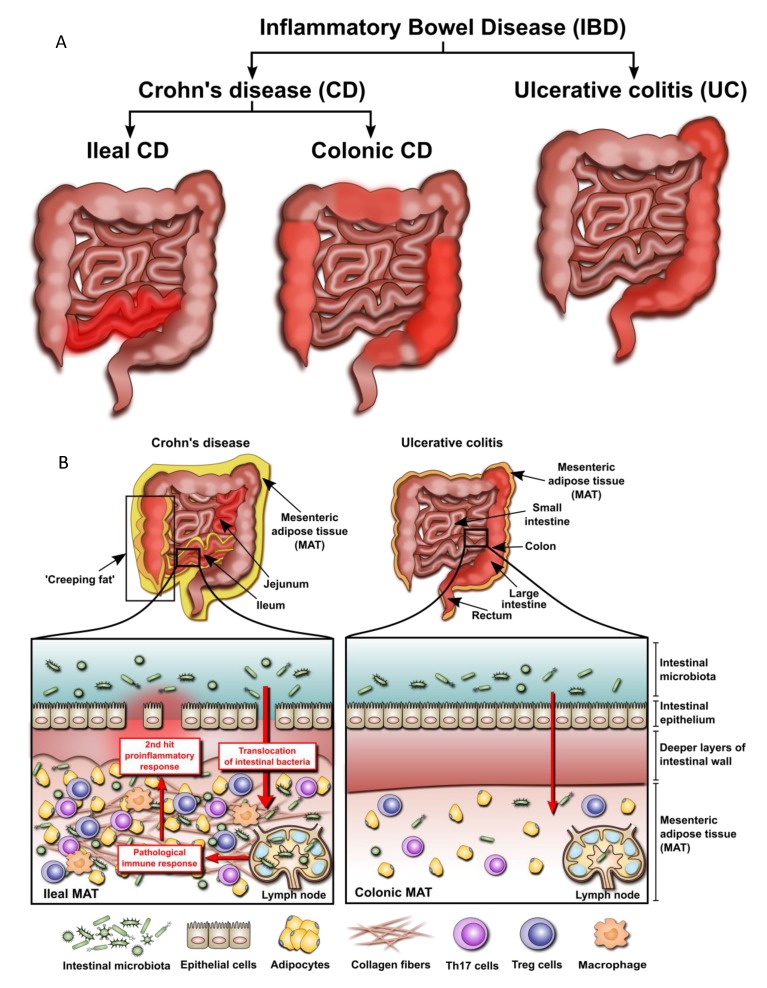
(**A**) Location of lesions in different forms of IBD. (**B**) Postulated differences in the involvement of mesenteric adipose tissue in IBD with ileal and colonic lesions. Ileal inflammation compromises the integrity of the intestinal epithelial barrier leading to translocation of altered intestinal microbiota into mesenteric fat and lymph nodes. Interaction of adipocytes with gut bacteria results in adipocyte hyperplasia, induction of proinflammatory genes and secretion of chemokines attracting various leukocyte populations. The accumulation of pathogenic bacterial species in mesenteric lymph nodes drives the immune response resulting in persistent inflammation in the mesenteric adipose tissue. This aggravates the destruction to the adjacent ileal wall, which further impairs the intestinal barrier and allows more gut bacteria to translocate to the mesentery. The resulting “vicious circle” fuels inflammation and leads to fibrosis. The translocation of intestinal microbiota during colonic inflammation appears to be less pronounced leading to only a moderate exposure of the mesentery to bacteria. As a result, adipocytes do not significantly amplify the inflammatory response so that there is no additional “hit” to damage the intestinal wall.

Zulian et al. [55] compared omental and mesenteric fat from IBD patients (UC, *n* = 11; CD, *n* = 11) with respect to adipocyte morphology, gene expression profiles and the presence of bacteria. Tissue from UC was observed to be less inflamed and contained fewer bacteria than that from CD. Interestingly, when preadipocytes isolated from the omentum of IBD patients were challenged with *Enterococcus faecalis* in vitro, they responded with a significant increase in proliferation. Altogether, these findings indicate that “creeping fat” contributes particularly to ileal CD rather than colonic disease. These results correspond to the distinctive disruption of the ileal intestinal epithelial barrier in CD. It may enable translocation of enteric bacteria to the mesenteric lymph nodes and adipose tissue resulting in its reactive hypertrophy and adipocyte proliferation. It appears, however, that bacterial translocation must be followed by a “second hit” to trigger a full-blown inflammatory reaction in the ileum (Figure 3).

### 2.4. Adipocytokines

Adipocytokines are adipocyte-derived mediators with endocrine, paracrine and autocrine activity. From over 50 adipocytokines identified to date [7], several have been linked to IBD. Their proposed role in the pathogenesis of IBD is outlined below.

#### 2.4.1. Leptin

Leptin is a 16-kDa peptide produced predominantly by adipocytes in proportion to body fat mass [56]. Its primary endocrine function is to regulate the appetite by signaling satiety to the hypothalamus [57]. Though rare, congenital leptin deficiency in humans leads to impaired T cell proliferation and cytokine release, and increased childhood mortality due to susceptibility to infections. These effects can be reversed by leptin supplementation [58,59]. Leptin exerts strong proinflammatory effects by synergizing with TNFα to activate macrophages [60] and generate reactive oxygen species in neutrophils [61]. It also regulates T-helper cell polarization [62], increases naïve T-cell proliferation [63] and interferon-gamma (IFN-γ) production by memory T cells [64]. In mice, intra-rectal administration of leptin results in NF-κB-mediated colitis with epithelial monolayer damage and neutrophil activation [65]. Consequently, leptin deficiency protects mice against DSS- and TNBS-induced colitis [56].

Measurements of leptin in human IBD produced mixed results. The majority of studies reported no difference in serum leptin between CD patients and healthy controls [45,66,67,68]. In pediatric populations, there was no difference in serum leptin both between CD patients and the controls, as well as between UC and CD patients [68,69]. However, interpretation of the results from these or similar studies is difficult as the analyzed populations often differ in terms of treatment received. Moreover, the data from treatment-naïve individuals is not always available and the control groups are not homogenous as they may include healthy individuals, IBD patients in remission or patients with gastrointestinal diseases other than IBD. Interestingly, analyses of leptin mRNA in diseased tissues have uniformly shown an increase in leptin expression in both UC [44,70] and CD [44,71]. This may suggest that upregulation of leptin in IBD is seen locally rather than systemically and local leptin acts by exerting autocrine and paracrine effects. Such a scenario is supported by data from murine models of colitis [72].

#### 2.4.2. Adiponectin

Adiponectin is a protein secreted almost exclusively by adipocytes [73,74] and accounts for almost 0.01% of all circulating protein. Low adiponectin levels have been associated with obesity [75], insulin resistance and type II diabetes [74]. It has anti-inflammatory, vasculoprotective and insulin-sensitizing effects [76]. Adiponectin has been linked to autoimmune and inflammatory conditions including Behcets’ disease [77], systemic sclerosis [78], psoriasis [79] and IBD.

During IBD, systemic levels of adiponectin do not change consistently, while tissue adiponectin expression appears to increase [71,80]. However, a more recent study found decreased mucosal expression of adiponectin in patients with active ileocaecal CD compared to patients with normal distal ileum [81].

Adiponectin exists in multiple isoforms differing in molecular weight [81]. Their respective roles are still poorly defined, but it appears that relative ratios of these isoforms may be more important for biological activity than an absolute concentration of a single molecule [81,82]. To our knowledge, no study has assessed the role of specific adiponectin isoforms in IBD.

#### 2.4.3. Resistin

Although initially identified in adipocytes, resistin is primarily expressed by macrophages both within and outside of adipose tissue [83]. It exhibits a strong proinflammatory activity by upregulating IL-6 and TNFα expression via the NF-kB signalling pathway [84]. Studies in IBD uniformly reported on elevated levels of resistin compared with healthy controls. However, there was no apparent difference in serum resistin between IBD and other diseases characterized by chronic inflammation, including non-alcoholic fatty liver disease, diverticular disease and colorectal cancer [85,86]. This may indicate that serum resistin is a non-specific marker of inflammation, which is indirectly confirmed by a decrease in resistin levels observed following anti-TNFα therapy both in IBD [87] and in rheumatoid arthritis [88].

#### 2.4.4. Visfatin

Visfatin is also known as pre-B cell colony enhancing factor (PBEF) or nicotinamide phosphoribosyltransferase (NAMPT). Although initially reported to be produced preferentially by VAT [89], it is now known to be secreted by other cell types as well [90]. Intracellularly, visfatin catalyzes the salvage pathway for nicotinamide adenine dinucleotide (NAD), a key co-enzyme in cell energy-consuming processes such as inflammation and cell proliferation [91]. Circulating visfatin has been found to be significantly elevated in both CD and UC patients compared with controls.

Given its functions in cellular metabolism, the observation of increased visfatin expression in foci of active inflammation in IBD was not surprising [92,93]. Starr et al. found a higher expression of visfatin in colonic biopsies from 99 children with IBD naïve to therapy [92]. In addition, Moschen et al. reported on increased visfatin tissue expression in UC and CD [93]. They also found that visfatin upregulated the production of IL-1, IL-6, IL-10, and TNF-α by monocytes. Moreover, visfatin was found to act as a potent chemotactic factor for monocytes and B cells and an activator of antigen presenting cells, phagocytes, and T cells [93]. These multiple functions make visfatin a potential therapeutic target. A recent pre-clinical trial of FK866, an inhibitor of intracellular NAMPT, showed an improved course of experimental colitis with a shift in macrophage sub-populations toward an anti-inflammatory M2 phenotype [94]. In human IBD-derived lamina propria mononuclear cells, FK866 diminished cytokine release to the extent comparable to that of dexamethasone and infliximab. Visfatin inhibitors were also investigated in phase I trials but were not successful due to dose-limiting toxicity and systemic activity [95].

#### 2.4.5. Chemerin

Chemerin is a proinflammatory cytokine implicated in adipocyte differentiation and metabolism, insulin resistance and blood pressure control [96,97]. It is secreted as an inactive molecule, which is rapidly activated by neutrophil-derived proteases at sites of inflammation [98]. Chemerin has been associated with multiple inflammatory conditions including rheumatoid arthritis and psoriasis, as well as ovarian and liver cancers [99]. It exhibits a chemokine-like activity, and can provide a link between innate and adaptive immunity through recruitment and activation of antigen presenting cells [97]. Administration of exogenous chemerin aggravated the severity of DSS-induced experimental colitis by decreasing the numbers of anti-inflammatory M2 macrophages and by increasing the production of the proinflammatory cytokines TNFα, IL-6 and IFN-γ [99]. Local levels of chemerin correlated with the severity of colitis in mice [100]. Correspondingly, chemerin mRNA expression in tissue biopsies from patients with UC correlated with disease activity [99]. In contrast, studies on systemic levels of chemerin in IBD have produced mixed results. Compared with healthy controls (*n* = 80), a large population of IBD patients (CD, *n* = 230; UC, *n* = 80) was found to have elevated concentrations of circulating chemerin [101]. This observation was confirmed by some [102] but not all studies [103].

#### 2.4.6. Ghrelin

Secreted by gastric endocrine cells, ghrelin reduces differentiation of pre-adipocytes to adipocytes through attenuation of peroxisome proliferator-activated receptor-gamma (PPAR-γ) [104]. It antagonizes leptin by inhibiting leptin-induced proinflammatory responses in macrophages and T cells, reducing the expression of proinflammatory cytokines (including TNFα, IL-1β, IL-6, and IL-8) and decreasing the expression of leptin in the gastrointestinal tract [105]. Compared with healthy controls, the expression of ghrelin in the colon is upregulated both in CD and in UC [66,106,107,108,109]. As with other adipocytokines, systemic levels of ghrelin do not change consistently in IBD. In TNBS-induced murine colitis, intraperitoneal administration of exogenous ghrelin improved recovery without affecting PPAR-γ expression [106]. Ongoing clinical trials are assessing ghrelin mimetics as novel prokinetic agents for gastrointestinal motility disorders [110,111] and as appetite stimulants for cachexia. The potential effect of ghrelin on the course of IBD remains to be explored.

#### 2.4.7. Other Notable Mentions

Serum concentration of vaspin (visceral adipose tissue derived serpin) is associated with obesity and insulin resistance in humans [102]. A single study in IBD (CD, *n* = 67; UC, *n* = 48) showed no difference in vaspin between IBD patients and healthy controls [102]. Retinol binding protein 4 (RBP-4), another mediator linked to the metabolic syndrome [112], has been reported to be elevated in IBD [65] and inversely correlated with disease activity [113]. Omentin-1 is expressed predominantly in omental tissue and thought to exert anti-inflammatory activity by inhibiting TNFα [114]. Omentin-1 was found to be decreased in obesity, type 2 diabetes, coronary artery disease and more recently in IBD [114,115]. Lu et al. observed lower concentrations of omentin-1 in serum from patients with active CD compared with patients with CD in remission or healthy controls. In these patients, ometin-1 levels correlated inversely with disease activity, as reflected by CDAI, TNFα and C-reactive protein (CRP) [115]. Moreover, omentin-1 mRNA expression was found to be reduced in colonic tissue from active CD [115].

In summary, there are increasing clinical and experimental data showing that the adipose tissue, especially VAT, is involved in IBD. This is achieved by participating in immune responses to gastrointestinal microbiota and by secreting a number of key mediators with inflammation-modulating activities. Thus, adipose tissue presumably affects both the disease course and therapeutic outcomes.

## 3. The Impact of Adipose Tissue on Clinical Course and Therapeutic Outcomes in IBD

### 3.1. Adipose Tissue and Clinical Course of IBD

In spite of the previously described inconsistencies, there is a reasonable basis to suggest that VAT can significantly modulate the course of inflammation in IBD. Thus, it can be hypothesized that the measurement of MAT/VAT volumes or MAT-derived mediators can reflect IBD activity. Sheehan et al. [19] were among the first to demonstrate that the presence of fat-wrapping in the intestines is associated with ulceration, stricture formation, increased wall thickness and transmural inflammation in CD. Since then, several studies have confirmed this phenomenon to correlate with CD activity. Li et al. demonstrated mesenteric fat (quantified through computed tomography (CT) images) to correlate significantly with disease activity as measured by CDAI and CRP [20]. Likewise, hypertrophy of MAT (as assessed by ultrasound) correlated with clinical and biochemical parameters of CD, including formation of internal fistulas, CRP and CDAI [116]. The mesenteric fat index (MFI), i.e., the ratio of visceral to subcutaneous fat, has been proposed as a biomarker of complicated CD [117,118]. Erhayiem et al. demonstrated that an MFI of 0.29 identified patients with complex CD with 93% sensitivity and 81% specificity. Visceral accumulation of fat was higher in cases of fistulizing and stenotic disease [118]. Another parameter that can reflect the relationship between VAT and CD activity is the VAT/total fat mass (FM) ratio. Buning et al. [119] showed that high VAT/FM ratio was associated with B2 and B3 CD behavior, according to the Montreal classification. Moreover, this ratio appeared to have also some predictive value, since it correlated with shorter remission times among female CD patients.

Furthermore, the analysis of 482 CD patients from the PRISM database showed that the volume of visceral fat correlated with the risk of developing penetrating disease. Individuals from this population in the highest quartile of VAT volume had an odds ratio as high as 2.02 for surgical intervention compared to individuals in the lowest quartile, which remained even after adjusting for genetic susceptibility [120].

A similar trend was evident in pediatric CD patients. Uko et al. [117] showed that children with higher VAT volumes, as measured by abdominal CT, had an increased risk of developing fistulizing and fibrostenotic disease. Moreover, these children were hospitalized more often, required earlier surgery and had higher disease activity scores at diagnosis. Through magnetic resonance (MR) fat quantification, Frivolt et al. [121] confirmed that the expansion of intra-abdominal adipose tissue was associated with increased complexity of the disease and its duration.

Additionally, visceral adiposity was found to be an independent predictor of post-operative morbidity in CD patients undergoing bowel resection [122]. Patients with a surface area of visceral fat >130 cm^2^ (on cross-sectional CT images at the level of L3) required longer and more extensive surgery, lost more blood and were more likely to experience post-operative ileus. In this population, the odds ratio for overall postoperative complications was 2.69 (95% confidence interval 1.09-6.62) [123]. Furthermore, a sub-study [124] of the POCER (Post-Operative Crohn′s Disease Endoscopic Recurrence) trial revealed that after adjusting for height, all patients with a VAT/height^2^ ratio > 1.5 times the gender-specific mean experienced endoscopic recurrence of the disease at 18 months (relative risk 2.1, 95% confidence interval 1.5–3.0, *p* = 0.01); hence, visceral adiposity was an independent risk factor for unfavorable CD course after surgery. This conclusion was further supported by Li et al., who found that a large VAT area and high MFI correlated with endoscopic scores and disease recurrence [125]. Moreover, multivariate analysis indicated that a VAT area above the median was predictive of clinical CD recurrence after surgery (hazard ratio 2.63, 95% confidence interval 1.03–6.47). These data collectively suggest that the expansion of VAT and, hypothetically, its high metabolic activity can fuel chronic inflammation in the gastrointestinal tract.

### 3.2. Adipose Tissue and IBD Therapy

Azathioprine is the first-line treatment option for moderate-to-severe IBD [126]. The standard dosing regimen is based on patient’s weight, while accurate measurements of drug metabolites are performed usually only when patients do not respond to therapy. In this respect, there exists an association between the level of 6-thioguanine nucleotide (6-TGN), an azathioprine therapeutic metabolite, and clinical remission [127]. Holt et al. [128] used cross-sectional CT imaging to investigate if body composition could provide more accurate means of achieving therapeutic levels of 6-TGN. They found no relationship between therapeutic levels of 6-TGN and subcutaneous or visceral fat, suggesting that the distribution of fat has little impact on thiopurine therapy.

In contrast, adiposity has been linked with suboptimal responses to biologic therapies [129]. A single-center retrospective study of UC patients (*n* = 160) treated with biologic agents found that an increase in BMI by 1 kg/m^2^ increased the risk of treatment failure and surgery/hospitalization by 4% and 8%, respectively [130]. However, the largest analysis published to date, with pooled data (*n* = 1205) from four large randomized clinical trials (ACCENT-I, SONIC, ACT-1, ACT-2), showed no inferior response to infliximab by obese patients [131]. There was no apparent association between obesity (as defined by BMI ≥ 30 kg/m^2^) with clinical course or mucosal healing. This was the case for both CD and UC, as well as for induction and maintenance therapy. The lack of difference could be related to the weight-adjusted dosing regimen of infliximab. Importantly, the study did not differentiate between subcutaneous and visceral adipose tissue, as this cannot be reliably estimated by BMI [132]. Therefore, a recent retrospective study of 97 CD patients undergoing infliximab induction therapy investigated specifically the relationship between visceral adiposity (MFI obtained from CT images) and the rate of mucosal healing [133]. The lower content of visceral fat but not subcutaneous fat, was demonstrated to be independently associated with mucosal healing. This suggests that the response to some biologic therapeutics may be modulated by body fat distribution. In this respect, not only will it be interesting to confirm the impact of body fat on the efficacy of agents applied at doses corrected for body mass, such as infliximab, but to also determine the role of adipose tissue distribution on agents applied at fixed doses such as adalimubab. In our own study, we used MR enterography (MRE) to monitor CD patients on infliximab therapy [134]. MRE has been previously validated as a non-invasive method to assess CD activity [134]. We observed that reduced fat wrapping corresponded with improved clinical and biochemical status. While this may support the proposed role of “creeping fat” as a reactive inflammatory response, no study to date has evaluated the effects of anti-TNFα therapy on local adipocytokine expression. Frivolt et al. [135] measured circulating adipocytokines during induction therapy with infliximab in pediatric CD patients (*n* = 18). They found that adiponectin increased significantly above baseline after 2 weeks but fell below the baseline at 14 weeks. Another study of CD patients (*n* = 20) [136] showed a significant increase in circulating leptin as early as 1 week post-infliximab induction, before a weight gain could be clinically demonstrated. These observations may suggest that changes in adipocytokine profiles after TNFα blockade could impact mesenteric fat regression. However, the exact mechanism of this association needs to be elucidated.

At present, there are no therapeutic strategies in IBD that would specifically target visceral or mesenteric fat. In recent years, PPAR-γ has been implicated in the adipocyte hyperplasia in the mesenteric adipose tissue [137]. PPAR-γ was found to be upregulated in the MAT of CD patients and not in SAT or healthy controls [21,138]. Stimulation of PPAR-γ is multifactorial and can occur as a result of obesity, high dietary intake of fatty acids, as well as activation of TLR4 by bacterial products [139], which all may contribute to the “creeping fat” phenomenon. The pharmacological blockade of PPAR-γ signaling in CD may, however, be problematic, because PPAR-γ is essential for the maintenance of epithelial expression of a beta-defensin *DEFB1* that protects against mucosal adherence of certain microorganisms, and its expression was found to be reduced in colonic CD [140].

Unlike in CD, PPAR-γ signaling in UC appears to be impaired and correlates negatively with the endoscopic severity of the disease [140]. Administration of PPAR-γ agonists in experimental colitis led to significantly better preservation of tissue histology [141]. In humans, randomized clinical trials showed a significant reduction in UC activity with rosiglitazone, a PPAR-γ agonist [142]. Its routine use, however, may be problematic due to concerns about its cardiovascular safety profile [143]. Mesalazine (or 5-aminosalicylic acid; 5-ASA), a first-line therapy for mild-to-moderate UC, can also act as a ligand for PPAR-γ, with current research seeking to develop 5-ASA analogues with even stronger affinity for PPAR-γ [144]. A novel PPAR-γ modulator, GED-0507-34 Levo, has shown promising results in ameliorating colitis and intestinal fibrosis [145]. However, the phase II SEGMENT trial was terminated prematurely due to recruitment issues [146].

## 4. Conclusions

Far from being an innocent bystander, visceral adipose contributes to the pathogenesis of IBD and determines disease severity and outcomes. It exhibits proinflammatory and immunoregulatory properties driven by changes in local cytokine and hormone environment. However, the exact mechanisms by which visceral adiposity in IBD mediates these effects remain obscure, largely due to difficulties in separating the overlapping functions of SAT and VAT, of which only the latter appears to be relevant to IBD. Therefore, further research efforts are required if visceral and mesenteric fat is to become a therapeutic target in IBD.
